# Experimental evolution of fungal pathogens as a tool to unravel host adaptation and virulence

**DOI:** 10.1371/journal.ppat.1014149

**Published:** 2026-04-24

**Authors:** Paul Mathias Jansen, Bernhard Hube, Sascha Brunke

**Affiliations:** 1 Department of Microbial Pathogenicity Mechanisms, Leibniz Institute for Natural Product Research and Infection Biology, Jena, Germany; 2 Institute of Microbiology, Friedrich Schiller University, Jena, Germany; 3 Cluster of Excellence Balance of the Microverse, Friedrich Schiller University Jena, Jena, Germany; University of Maryland, Baltimore, UNITED STATES OF AMERICA

Microbial pathogenesis has evolved over time and continues to evolve. Some virulence factors are selected for by their contribution to survival in the environment and only accidentally enable virulence in susceptible hosts [1]. Other pathogens co-evolve(d) with hosts to aid their survival and propagation. These processes could be described as a gigantic and, arguably, successful natural experiment, which has been ongoing for millions of years.

Evolution can be leveraged to understand microbial virulence when recreated under laboratory conditions. Such evolution experiments (or “adaptive laboratory evolution”) are still rarely used compared to other approaches. However, their strength is that they test functions throughout the genome without the need of any initial hypothesis.

## The rationale behind evolution experiments

Model organisms like *Escherichia coli* are often used to understand the evolution of genomic and phenotypical changes in well-controlled experimental environments [2]. One of the most famous examples is the long-term experimental evolution experiment (LTEE) by Lenski *et al.*, in which *E. coli* has been cultivated in a glucose-limited environment for decades, with surprising outcomes [3]. Our ability to analyze the results of such evolution experiments has massively expanded with the advent of next-generation sequencing [4] and CRISPR-Cas9 [5].

Experimental evolution can also be used to investigate eukaryotic organisms, mostly *Saccharomyces cerevisiae*. Such experiments have revealed the astonishing adaptability of fungi and provided insights into the basis of virulence in pathogenic fungi.

Considering the large number of fungal species, only few are successful human pathogens that cause life-threatening diseases [6]. These fungi either come from the environment or are commensal members of the host’s microbiome, which only cause disease in immunocompromised or otherwise susceptible individuals, and novel pathogenic fungi emerge due to climate changes and increased opportunities to infect due to natural disasters [7].

An early experimental evolution of a pathogenic fungus by Franzot *et al.* revealed that *Cryptococcus neoformans* became less virulent by continuous laboratory cultivation [8]. Since then, many studies focused on the evolution of antifungal resistance (reviewed in Jacobs *et al.* [9]). We will here instead explore a—necessarily limited—selection of studies that exemplify possible adaptation to the host.

## The evolution of stress resistance and its consequences

Fungal pathogens need to adapt to many stressors to successfully colonize their prospective hosts. For example, they face shifting levels of carbon dioxide or oxygen in distinct niches, which is comparatively easy to simulate in the laboratory, but can yield highly interesting results: Under high CO_2_ levels, environmental *C. neoformans* isolates quickly adapted by loss-of-function mutations in the virulence and carbon metabolism regulator gene, *AVC1* [10], strikingly recapitulating mutations found in patients with relapsed cryptococcal meningitis. A laboratory strain of *Aspergillus fumigatus* quickly adapted to hypoxia, which is found in lesions during invasive pulmonary aspergillosis. The evolved strain not only showed increased growth rates, but also higher virulence in a murine model [11]. Fungal pathogens are also often exposed to oxidative stresses in the host, for example, by macrophages. Huang and Kao investigated the resistance of *Candida glabrata* [12] to increasing H_2_O_2_ concentrations, where it developed enhanced H_2_O_2_ detoxification and resistance to oxidative stress by several mutations known to alter fungal membrane lipid composition and increase detoxification.

Opportunistic environmental pathogens are confronted with a significant temperature increase in the human body. Adaptations to higher temperatures can also have other advantages: The environmental fungal pathogen *Cryptococcus deneoformans* developed resistances against 5-fluoroorotic acid when passaged through an animal [13]. *In vitro* evolution experiments showed that incubation at 37 °C alone resulted in increased resistance, caused by inactivation of *URA3* and *URA5* by transposable elements (TE) insertions. A later study revealed genome-wide and specific TE activation at 37 °C [14], where some TEs integrated into intergenic regions affecting gene expression and others into genes with specific binding motifs. This temperature-dependent increase in mutation rate allows adaptation to mammalian body temperatures and the development of pathogenicity.

A stepwise increase from 30 to 35 °C also affected the virulence of a temperature-sensitive *C. neoformans* strain [15]*.* It evolved tolerance to temperatures even exceeding the evolution experiment’s range. This was linked to unexpected mutations found independently in different lineages, e.g., in the hexose transporter Hxt1. This was interestingly accompanied by increased fluconazole resistance, but some lineages lost the ability to titanize, an important virulence attribute of *C. neoformans* [15]. Studies like this demonstrate the power of evolution experiments to reveal unforeseen evolutionary paths.

Fungi also have to counter chemical assaults in the host, including high levels of copper within phagosomes. Handelman *et al.* showed how *A. fumigatus* adapted to increasing copper levels, by mutations in several novel genes, with the trade-off of reduced growth in absence of copper [16]. Interestingly, these mutations did not alter virulence in mice. In another study, adaptation to increasing ethanol concentrations reduced the cell size of *C. albicans* and increased its ethanol tolerance, but surprisingly decreased its susceptibility to the antifungal fluconazole [17]. The underlying mutations, including a trisomic chromosome R, are often found in fluconazole-resistant isolates. Evidently, adaptation to noxious chemicals can increase drug tolerance and even virulence of fungal pathogens.

## Adaptive strategies to the host’s nutritional landscape

While colonizing humans, microbes are confronted with changing availabilities of nutrients and their uneven distribution in different niches. Metabolic flexibility is therefore paramount for the ability of pathogens to colonize and persist.

A central nutrient for survival in the host is inorganic phosphate. In an evolution experiment, *C. albicans* mutants lacking all phosphate transporters were grown under limited inorganic phosphate. The lineages not only regained fitness, but also gained resistance against some common stressors [18]. Carbon sources also affect the evolutionary course of fungi: In one study, *Candida auris* grew as filaments after passaging through a mammalian host [19]. Deng *et al.* were able to replicate this phenotype by simple growth on glycerol as sole carbon source [20], resulting in improved fatty acid β-oxidation and skin colonization. Adaptation to specific nutrients can also result in increased virulence, as was shown by Lange *et al.* [21]: Continuous exposure of environmental *C. albicans* isolates to galactose increased growth with this dietary sugar. The evolved strains surprisingly also exhibited antifungal resistance and increased human epithelial damage. This shows that host-mimicking experimental conditions can reveal possible evolutionary trajectories of within-human adaptation.

On the other hand, growth in rich laboratory media can reveal basal genome changes that occur by continuous culture alone. An evolution experiment with a rich medium targeted the dynamic subtelomeric regions of the *C. albicans* genome [22]. Sixteen distinct subtelomeric recombination events were found that altered the telomere-associated (*TLO*) gene repertoire, in one case even creating a novel *TLO* sequence encoding a new Tloα variant protein. This supports the notion that subtelomeres are a hotspot for gene evolution due to higher rates of recombination, gene duplications, and mutations, which influence the virulence and adaptability of fungi [23]. In a recent study clinical isolates of *C. albicans* adapted to rich media showed decreased resistance to multiple stressors and a reduced ability to colonize mice, stemming from lower TOR activity [24]. These are important observations for the applicability of laboratory experiments to “real life”, since laboratory strains have often been cultivated in rich media for many generations.

Genomic alterations allow the adaptation of fungal pathogens to their host, and this happens at an increased rate with the mutator phenotype. The mechanisms behind this were identified in *C. neoformans* by experimental evolution [25]: Deletion mutants of mismatch repair pathway genes (*MSH2*, *MLH1*, and *PMS1*) incubated on rich media plates rapidly improved their ability to grow at higher temperatures, withstand oxidative stress, resist antifungal chemicals, and, remarkably, proliferate faster in a mouse model, while only one mutant (of *PMS1*) showed attenuated virulence. This emphasizes the advantages of genetic flexibility during host adaptation and infection.

## Evolution to evade phagocytotic predation

Evading or overcoming the host’s immune system is indispensable for successful fungal pathogens. Evolution experiments on this subject have mostly targeted interactions with macrophages (as part of first-line immune defense), but evolving with their natural predators, amebae, also provided important insights [26].

Adaptation to macrophages by continuous coculture has shed light on *C. glabrata*’s immune evasion [27]. A macrophage-passaged lineage developed pseudohyphae-like growth that increased fitness within macrophages and allowed fungal cells to damage phagocytes from within, combined with higher virulence in a murine systemic infection model. All these phenotypes arose from a single nucleotide mutation in the chitin synthase gene, *CHS2*, strikingly demonstrating how easily increased virulence can arise. A similar filament-capable morphology was discovered by Yue *et al.* [19] in *C. auris*, which was also thought to be incapable of filamentation, after systemic infection of mice.

A comparable experiment revealed another layer of the yeast-to-hyphae transition in *C. albicans*, which is important for escaping macrophages [28]. When a yeast-locked mutant was continually passaged with murine macrophages, the evolved strain regained the ability to filament in response to different triggers and to escape from macrophages and invade into epithelial cells. It even regained most transcriptional hallmarks of filaments despite lacking two central transcription factors. As with *C. glabrata*, this phenotype was linked to a single mutation. Clearly, even major morphological changes can be triggered and shaped by evolutionary pressures, which suggests several possible ways for the emergence of dimorphic fungi like *C. albicans*.

Ameba and macrophages share many similarities, and ameba predation can serve as an accessible model for macrophage phagocytosis in evolution experiments. Indeed, amebae are thought to “train” environmental fungal pathogens for immune evasion [29], although, of course, much of this data was gained with laboratory-adapted *Acanthamoeba castellanii*, and not in wild ameba that come in a broad range of varieties. In a recent study, the adaptation of *C. neoformans* to ameba and macrophage predation was directly compared and found to generally protect best from the original predator [30]. The enhanced growth of one lineage was traced to a single nucleotide change in the adenylyl cyclase gene, *CAC1*. This mutation increased survival in macrophages, but, somewhat counterintuitively, decreased mortality of mice after infection—potentially because other virulence-related phenotypes, like the cell size plasticity, were negatively affected [30]. This shows that fungal pathogens can evolve rapidly to avoid predation by isolated macrophages but may specialize to the detriment of overall pathogenicity. How this translates to a setting like sub-niches in the human body, where the selection pressure is more complex than a single cellular predator, remains an open question.

Evolution experiments with the ameba *A. castellanii* were also conducted with *C. albicans*, which can be found (albeit rarely) in the environment [31]. *C. albicans* became more filamentous, virulent, and showed better survival within ameba – mirroring the strong selection for filamentation by macrophage exposure [28]. The ameba-evolved strains showed higher resistance to many stresses, including heat and in some cases even fluconazole. Such increase in virulence was already observed in one of the earliest evolution experiments involving ameba [32]: When *C. neoformans* was passaged through the ameba *Dictyostelium discoideum*, it evolved larger capsules and faster melanization, both important virulence factors. Together, these experiments suggest that ameba predation is not only useful to model survival within macrophage, but may even drive virulence of environmental opportunistic pathogens in real-life, a concept known as “environmental virulence school” [26].

## Adaptations in animal models

Animal colonization and infection models provide the closest approximation for the challenges in a human host, and environmental pathogens can be transiently associated with animals. *In vivo* evolution experiments can therefore allow us to understand adaptations to complex host traits.

Basic parameters, as the rate and type of mutations in *in vivo* environments and how they differ from laboratory media, were investigated early on by Forche *et al.* with *C. albicans* [33]*.* They used passages through a systemic infection mouse model and compared this to growth in rich media*.* While the growth rates of mice-passaged *C. albicans* were lower, *in vivo* evolution led to more recombination and chromosome-level changes, and, importantly, more phenotypic variation. This shows that the genetic mechanisms driving adaptations can differ between the host and other environments.

Although *C. albicans* is a pathogen, the fungus predominantly colonizes mucosal surfaces as a harmless commensal. Forche *et al.* studied its evolution as a colonizer of the oral cavity of mice [34]. Again, they found a (here 100-fold) higher frequency of loss of heterozygosity (LOH) events *in vivo* compared to *in vitro*, starting on the first day, and frequently a trisomy of chromosome 6, which harbors many virulence-related genes. Surprisingly, partial or even complete haploidization of the normally diploid *C. albicans* was also observed, which reverted quickly on standard media, suggesting an advantage specifically in the oral cavity—once more showcasing the speed of evolutionary processes in changing environments. In a follow-up study on the advantages of trisomies (specifically of chromosome 6) for colonization [35], trisomic strains colonized the murine oral cavity like their diploid progenitor strain, but triggered less weight loss and inflammation *in vivo*, and showed less adherence to and invasion into oral epithelial cells *in vitro*. Thus, these trisomies seem to provide an advantage for the commensal lifestyle of *C. albicans* in the oral cavity.

Ene *et al.* looked into genomic differences between evolved strains from systemic infections and commensal colonization, compared again to *in vitro* passaging [36]. Genomic changes were mostly base substitutions and short-tract LOH events in both *in vivo* and *in vitro* culture setups, and lineages passaged *in vivo* exhibited a higher mutation rate than *in vitro*. Specifically, chromosome 7 trisomies were found to confer a colonization advantage in the gastrointestinal tract of mice. Together with previous studies this emphasizes again that evolution in a living host elicits more genomic changes than *in vitro* growth.

An unexpected outcome was found when *C. albicans* was passaged repeatedly through the gut of antibiotic-treated mice [37]. These lineages consistently lost their ability to form hyphae, in stark contrast to the macrophage or ameba experiments described above. This loss of filamentation was often due to mutations in the same hypha-regulating transcription factors, which significantly increased competitive fitness in the murine gastrointestinal tract. Antibiotic treatment of the mice was required for this outcome, suggesting a selection pressure by the microbiome to retain hypha formation [37]. The gut-evolved lineages were also less virulent in a systemic mice infection model, in agreement with the view that bacteria of the microbiome exert pressure on *C. albicans* to retain its filamentation abilities and thus its virulence potential [38]—suggesting that traits that are useful for commensalism can also be advantageous during infection (“commensal virulence school”) [26].

Adaptations to different organs can also result in different virulence potentials: *C. albicans* that were passaged through the murine spleen *via* systemic infection became less susceptible to phagocytosis, but also less virulent in the mouse model. Likely, adaptation to the immune cell-rich spleen resulted in lineages that could persist in the host without killing it [39]. Passaging through the murine kidneys instead [40] did not measurably change *C. albicans* virulence. Existing adaptations to different organs (the kidneys are main target organs of *C. albicans* mice infections [41]) presumably led to these divergent outcomes.

In contrast to the reduced virulence often seen with *C. albicans*, environmental isolates of *C. neoformans* increased in virulence after passaging through mice [42]. A striking reduction in time to death was linked to increased expression of the fungal iron reductase gene, *FRE3*—a clear sign of adaptation to the iron-restricted host environment. These findings match one of the earliest fungal *in vivo* evolution experiments, in which *C. neoformans* also showed increased virulence after mouse passaging [43]. In another interesting study, researchers recapitulated the evolution of a strain from a cockatoo that caused an infection in its owner [44]. The cockatoo-derived strain was passaged twice through mice and re-isolated from either the lung or the brain. All mouse-passaged lineages gained larger capsules and increased survival with ameba. Significantly, all shared the same mutation with the patient’s strain, which was not originally present in the cockatoo strain. The authors posited that passaging through a mammalian host recapitulated the adaptation of the cockatoo-derived strain during the infection of the bird’s owner. Differences were found depending on the organ from which the evolved lineages were isolated: Only brain isolates additionally developed higher temperature tolerance and melanization (a key virulence factor) and were more virulent in a *Galleria* model. This agrees well with the organ-dependent evolutionary trajectories in the *C. albicans in vivo* evolution experiments described above, and overall, these studies demonstrate that *in vivo* studies in mice are well-suited to simulate the evolution of opportunistic pathogens in their human host.

Other laboratory animals have also been used in evolution experiments, since mouse experiments are often costly, time-consuming, and ethically challenging. The nematode *Caenorhabditis elegans* was used to investigate the effects of the host environment on the genomic stability of *C. albicans* [45]. Several laboratory strains and clinical isolates, either diploid or tetraploid and from different human body sites, were co-incubated with the nematodes or grown in laboratory media. Genome size changes and LOH occurred in all strains incubated with nematodes but depended on the genetic background and ploidy. For example, higher LOH rates were found in nematode-adapted laboratory and oral strains, but not in bloodstream isolates. All tetraploid strains showed rapid genome size reduction in the host, while genome reduction occurred *in vitro* to a lesser extent. The impact of tetraploidy on the virulence of *C. albicans* was investigated in a follow-up study [46]. Immunocompetent or immunocompromised *C. elegans* were infected with *C. albicans*, and in each round either the most virulent or a random *C. albicans* isolate was selected for the next infection. The first selection regime resulted in a rapid increase in virulence, while no change was observed by random propagation. Genome size reduction of the tetraploid strains was again observed in immunocompetent hosts, but much less in immunocompromised *C. elegans*. These findings stress how immune interactions are one of the most important evolutionary pressures fungal pathogens face within a host.

## Conclusions

**Fig 1:**
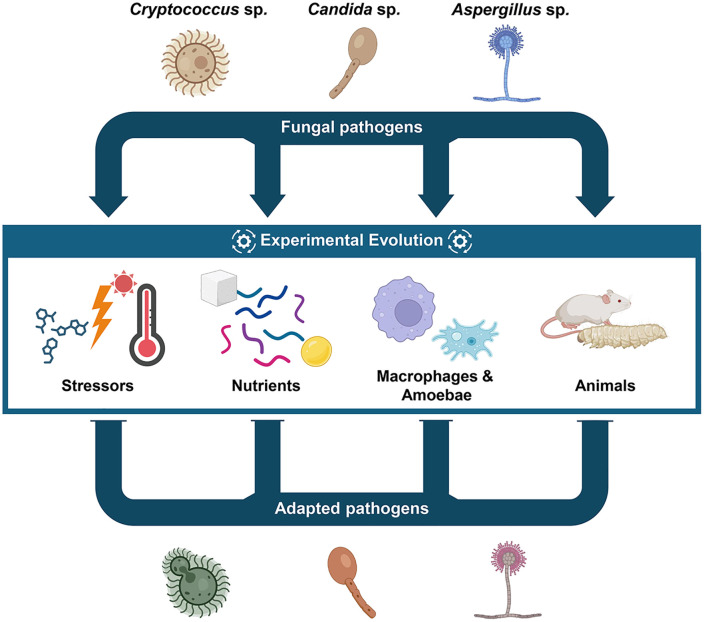
The different selection pressures that can be used in experimental evolution experiments to simulate host adaptation. Partially created in BioRender. Hube, B. (https://BioRender.com/fdptb0l) is licensed under CC BY 4.0.

We have compiled a nonexhaustive list of publications where experimental evolution was used as a powerful approach to investigate fungal pathogens (Fig 1) that does not require an *ab initio* hypothesis of the underlying mechanism. While experimental evolution has mostly been used to dissect resistance mechanisms against antifungals, this review demonstrates that it also has huge potential to investigate complex traits such as fungal virulence—with all due care to not overinterpret the outcome but rather see it as a glimpse into what is available as an evolutionary trajectory. From simple *in vitro* experiments with stresses or nutrients that can be found in the host to elaborate ones with host cells or whole animals as selection pressures, all the experiments described here provided surprising outcomes that taught us something new about the origins and mechanisms of virulence in pathogenic fungi. In a way, these experiments show us a bit of what is possible, but not necessarily all of what happens in the infinite evolution experiment that is real-life.

The success and increasing interest in this type of experiment is not least due to the development of easy and affordable whole genome sequencing methods, and transformation techniques like CRISPR-mediated gene editing. The former allows to investigate the fixation of beneficial alleles over time, even in parallel cultures, and the latter can be used to re-introduce the mutation into the parent genome to ascertain its contribution to the evolved phenotype. Many of the recent papers reviewed here made use of those techniques, and their number will likely rise significantly in the near future. As we have shown in this short review, experimental evolution has already provided important insights and will continue to be a powerful tool in medical mycology research.
